# Skin of Color Representation on Wikipedia: Cross-sectional Analysis

**DOI:** 10.2196/27802

**Published:** 2021-07-16

**Authors:** William Kim, Sophia M Wolfe, Caterina Zagona-Prizio, Robert P Dellavalle

**Affiliations:** 1 University of Colorado School of Medicine Aurora, CO United States; 2 Dermatology Service Rocky Mountain Regional VA Medical Center Eastern Colorado Health Care System Aurora, CO United States; 3 Colorado School of Public Health Aurora, CO United States

**Keywords:** skin of color, Wikipedia, dermatology, skin photographs, skin color, dermatology, eHealth, representation, SOC, skin conditions, photos, images, medical images

## Abstract

**Background:**

Wikipedia is one of the most popular websites and may be a go-to source of health and dermatology education for the general population. Prior research indicates poor skin of color (SOC) photo representation in printed dermatology textbooks and online medical websites, but there has been no such assessment performed to determine whether this discrepancy also exists for Wikipedia.

**Objective:**

The aim of this study was to investigate the number and quality of SOC photos included in Wikipedia’s skin disease pages and to explore the possible ramifications of these findings.

**Methods:**

Photos of skin diseases from Wikipedia’s “List of Skin Conditions” were assigned by three independent raters as SOC or non-SOC according to the Fitzpatrick system, and were given a quality rating (1-3) based on sharpness, size/resolution, and lighting/exposure.

**Results:**

We identified 421 skin disease Wikipedia pages and 949 images that met our inclusion criteria. Within these pages, 20.7% of images of skin diseases (196 of 949 images) were SOC and 79.3% (753 of 949 images) were non-SOC (*P*<.001). There was no difference in the average quality for SOC (2.05) and non-SOC (2.03) images (*P*=.81). However, the photo quality criteria utilized (sharpness, size/resolution, and lighting/exposure) did not capture all aspects of photo quality. Another limitation of this analysis is that the Fitzpatrick skin typing system is prone to subjectivity and was not originally intended to be utilized as a non-self SOC metric.

**Conclusions:**

There is SOC underrepresentation in the gross number of SOC images for dermatologic conditions on Wikipedia. Wikipedia pages should be updated to include more SOC photos to mend this divide to ameliorate access to accurate dermatology information for the general public and improve health equity within dermatology.

## Introduction

Wikipedia provides a broad range of information for the general public as the 8th most visited website in the United States and the 13th most visited website in the world [1]. Wikipedia may also be a go-to source of health education for the general population, including for information about dermatologic conditions. For example, the Wikipedia pages for psoriasis and leprosy have over 1 million views each, and one project to improve dermatologic Wikipedia pages found that 40 of these pages had over 10 million views combined [2]. Most of the pages dedicated to skin diseases have accompanying pictures to highlight these common skin pathologies, and the Cochrane Skin Wikipedia Initiative, supported by a board-certified dermatologist, has recently updated 80 dermatologic Wikipedia pages with information and photographs from Cochrane reviews [3]. However, many of the skin disease Wikipedia pages often do not offer adequate photo representation of skin of color (SOC) individuals. As more research on dermatologic conditions for SOC individuals emerges, it is clear that certain conditions such as melanoma, plaque-type psoriasis, and acne can present visually differently in people with darker skin compared to people with lighter skin [4]. These variable presentations can also alter treatment; for example, acne treatment may be based on expected hyperpigmentation levels [4].

Given these visual variations in skin disease presentations based on an individual’s skin color, adequate SOC photo representation on Wikipedia is important for the information to be applicable to and usable by people of all skin colors. Ensuring accurate skin disease photo representation contributes to health equity by allowing individuals of all skin colors to access relevant information. Therefore, the aim of this study was to investigate the number and quality of SOC photos included in Wikipedia’s skin disease pages and explore the possible ramifications of these findings.

## Methods

Skin diseases from Wikipedia’s “List of Skin Conditions” page (that either specified dermatology as a specialty in the article or were discussed in a separate dermatology textbook) were included in this study [5]. We followed the categorization system from Wikipedia’s “List of Skin Conditions” for the major categories listed in Table 1 and Table 2 [6]. Each category of skin condition contained multiple individual skin pages. For example, under the category acneiform eruptions, there were pages on neonatal acne and acne vulgaris, among others. Each page had varying amounts of information on the skin pathology, with some more extensive pages including signs and symptoms, causes, pathophysiology, diagnoses, management, prognosis, and epidemiology, in addition to photographs displaying the associated skin findings. In our review, we categorized these photographs into Fitzpatrick skin types, with Fitzpatrick scores of 1-3 deemed as non-SOC and Fitzpatrick skin types 4-6 deemed as SOC [7,8].

Three raters independently counted the images on each skin page, assigned the Fitzpatrick type, and scored the photo quality [7,8]. The raters were third-year medical students at the University of Colorado School of Medicine who were interested in dermatology, with all raters having a bachelor’s degree and one having a master’s degree. The photos were rated on a scale of 1-3, with 1 being poor quality, 2 being average quality, and 3 being excellent quality. Each photo was assessed for sharpness, size/resolution, and lighting/exposure. A photo received a score of 1 if it failed all three of these criteria, 2 if it had 2/3 criteria, and 3 if it met all three criteria. Any discrepancies in photo quality among the raters were discussed until a consensus was reached.

Our photo quality criteria were chosen as dermatology is an exceedingly visual specialty that requires clear images to accurately identify and interpret skin pathology. Black and white images, paintings and drawings, or images with ambiguous Fitzpatrick type were excluded, as were images unrelated to the skin disease. Some images appeared in more than one article, and these were counted more than once, as they were important in the context of each individual article. The quality and quantity of images were then compared between the SOC and non-SOC groups using the Student *t* test.

**Table 1:** Percentage of skin of color (SOC) to non-SOC photos on the Wikipedia list of skin conditions.

Skin condition	Non-SOC photos, n (%)	SOC photos, n (%)	Total number of photos
Acneiform eruptions	13 (81)	3 (19)	16
Autoinflammatory syndromes	3 (60)	2 (40)	5
Chronic blistering	11 (92)	1 (8)	12
Conditions of the mucous membranes	30 (91)	3 (9)	33
Conditions of the skin appendages	43 (78)	12 (22)	55
Conditions of the subcutaneous fat	8 (89)	1 (11)	9
Congenital anomalies	9 (82)	2 (9)	11
Connective tissue diseases	48 (92)	4 (8)	52
Dermal and subdermal growths	45 (66)	23 (34)	68
Dermatitis	32 (84)	6 (16)	38
Disturbances of pigmentation	12 (71)	5 (29)	17
Drug eruptions	11 (79)	3 (21)	14
Endocrine-related	7 (50)	7 (50)	14
Eosinophilic	2 (100)	0 (0)	2
Epidermal nevi, neoplasms, and cysts	45 (88)	6 (12)	51
Erythemas	11 (92)	1 (8)	12
Genodermatoses	31 (74)	11 (26)	42
Infection-related	146 (67)	71 (33)	217
Lichenoid eruptions	2 (40)	3 (60)	5
Lymphoid-related	11 (92)	1 (8)	12
Melanocytic nevi and neoplasms	36 (92)	3 (8)	39
Monocyte- and macrophage-related	3 (75)	1 (25)	4
Mucinoses	3 (75)	1 (25)	4
Neurocutaneous	11 (79)	3 (21)	14
Noninfectious immunodeficiency-related	2 (100)	0 (0)	2
Nutrition-related	0 (0)	4 (100)	4
Papulosquamous hyperkeratotic	12 (100)	0 (0)	12
Pregnancy-related	6 (86)	1 (14)	7
Pruritic	8 (67)	4 (33)	12
Psoriasis	15 (100)	0 (0)	15
Reactive neutrophilic	8 (100)	0 (0)	8
Recalcitrant palmoplantar eruptions	2 (100)	0 (0)	2
Resulting from errors in metabolism	10 (100)	0 (0)	10
Resulting from physical factors	65 (88)	9 (12)	74
Urticaria and angioedema	7 (100)	0 (0)	7
Vascular-related	45 (90)	5 (10)	50

**Table 2:** Average quality rating of skin of color (SOC) and non-SOC photos.

Categories	Average quality of non-SOC photos	Average quality of SOC photos
Acneiform eruptions	1.69	2.33
Autoinflammatory syndromes	1.67	2.5
Chronic blistering	2.27	2.0
Conditions of the mucous membranes	2.07	1.67
Conditions of the skin appendages	1.74	1.75
Conditions of the subcutaneous fat	2.0	2.0
Congenital anomalies	2.33	2.0
Connective tissue diseases	1.92	1.75
Dermal and subdermal growths	2.24	2.65
Dermatitis	1.78	1.5
Disturbances of pigmentation	1.5	2.6
Drug eruptions	2.36	2.67
Endocrine-related	2.43	2.67
Eosinophilic	1.5	N/A^a^
Epidermal nevi, neoplasms, and cysts	2.31	2.17
Erythemas	2.36	2.0
Genodermatoses	1.77	2.09
Infection-related	2.08	1.89
Lichenoid eruptions	1.5	1.33
Lymphoid-related	1.55	3.0
Melanocytic nevi and neoplasms	1.64	2.0
Monocyte- and macrophage-related	2.0	2.0
Mucinoses	1.67	2.0
Neurocutaneous	1.64	2.0
Noninfectious immunodeficiency-related	1.5	N/A
Nutrition-related	N/A	1.75
Papulosquamous hyperkeratotic	1.75	N/A
Pregnancy-related	2.0	1.0
Pruritic	1.38	3.0
Psoriasis	2.27	N/A
Reactive neutrophilic	2.25	N/A
Recalcitrant palmoplantar eruptions	2.0	N/A
Resulting from errors in metabolism	2.5	N/A
Resulting from physical factors	2.29	2.0
Urticaria and angioedema	2.43	N/A
Vascular-related	2.18	2.0

^a^N/A: not applicable.

## Results

We identified 421 skin disease Wikipedia pages and 949 images that met our inclusion criteria. Within these pages, 20.7% of images of skin diseases (196 of 949 images; s=1.52 cm) were SOC (Table 1) and 79.3% (753 of 949 images; s=2.02 cm) were non-SOC, representing a significant difference (*P*<.001); the s values are the standard deviations of the *t* tests. Lichenoid eruptions had the highest percentage of SOC photos (60%) with 3 out of 5 images being SOC images. Categories with no SOC representation included eosinophilic, noninfectious immunodeficiency-related, papulosquamous hyperkeratotic, psoriasis, reactive neutrophilic, recalcitrant palmoplantar eruptions, resulting from errors of metabolism, and urticaria and angioedema. The average quality for SOC images was 2.05 (s=0.79 cm) compared to 2.03 (s=0.75 cm) in non-SOC images (*P*=.81) (Table 2).

## Discussion

### Principal Findings

This study found significantly fewer SOC images compared to non-SOC images in the dermatology-related Wikipedia skin pages. There was no significant difference in photo quality between SOC and non-SOC photos.

### Limitations

This study highlights the discrepancies in the total number of SOC photos represented on Wikipedia’s list of skin conditions. However, our findings did not show a significant difference in the quality of SOC vs non-SOC photos. This may have been influenced by the small range of the rating scale (1-3) or the photo quality criteria utilized (sharpness, size/resolution, and lighting/exposure). If the rating scale was more granular, it may have allowed for more nuanced differences in photo quality to emerge between the SOC and non-SOC mean photo qualities. Additionally, other aspects of photo quality, including noise amount, noise pattern, and compression quality, may have led to differences in photo quality between SOC and non-SOC photographs. The study was also limited by the nature of the Fitzpatrick skin typing system, which was not originally intended to be utilized as a non-self SOC metric [9]. Therefore, some SOC individuals fell into our grouping of non-SOC (Fitzpatrick skin types 1-3), which may have influenced our results.

### Recommendations

Regardless of the quality of the photographs, there is underrepresentation in the total number of images for SOC dermatologic conditions on Wikipedia. Previous research has shown SOC photo underrepresentation in a wide range of resources, including printed dermatology textbooks [7], online websites such as VisualDx and Dermnet [7], and USMLE preparatory materials [8]. Alvarado et al [7] assessed the percentages of dark-skin (Fitzpatrick types 5 and 6) images across a variety of dermatologic resources [7]. DermNet NZ had 2.8% dark skin images, whereas VisualDx had 28.5% dark skin images [7]. In comparison, our study found 20.7% SOC images on Wikipedia (Fitzpatrick types 4-6).

Compared to websites such as VisualDx (ranked in position 113,182) and Dermnet (ranked in position 26,412), Wikipedia (ranked in position 8) has substantially more US internet traffic and engagement as evidenced by the listed rankings on the Alexa website [1]. Although VisualDx and Dermnet are well-known sources of dermatology information for the medical community, they may not be as well utilized by the general public. Wikipedia is arguably one of the main sources of dermatology information for the general public, and the discrepancies in SOC representation have a larger influence on the public’s perception of dermatologic disease and care compared to other dermatology resources previously reported in the literature. Possible ramifications of this discrepancy include decreased access to accurate information for SOC patients, skewed societal perceptions of how dermatologic conditions manifest in SOC individuals, inadequate treatment, and potentially poorer outcomes. 

Specific dermatology-related Wikipedia pages that need updating with more SOC photographs to reflect the higher rates in individuals with SOC include hyperpigmentation, acral lentiginous melanoma, melasma, pityriasis alba, acne, and atopic dermatitis [4,10,11]. Wikipedia’s “melasma” skin page has only one photograph highlighting skin pathology, and it is of an ambiguous Fitzpatrick skin type. Similarly, Wikipedia’s atopic dermatitis page has only one picture, and it is of a non-SOC individual. Potentially lethal skin diseases should also have their pages updated. For example, acral lentiginous melanoma is a dangerous skin pathology that disproportionately affects SOC individuals but has no SOC skin photographs on Wikipedia [4].

One skin page that did have a significant number of SOC photographs was “keloid” (under the dermal and subcutaneous growth category) with 20 of 26 photographs being SOC photos, which is more aligned with the higher rates seen in black patients [12]. The other Wikipedia skin pages should be updated similarly to more closely match population statistics in order to improve access to accurate information and potentially improve safety.

### Conclusion

Wikipedia pages should be updated to include more SOC photos. Given that Wikipedia is open to editing, more teams dedicated to updating the material information on SOC dermatology findings and presentations, particularly those supported by board-certified dermatologists, can help bolster the information available. Doing so will help mend the divide between SOC and non-SOC photos on Wikipedia’s dermatology pages and improve access to accurate dermatology information for the general public, thereby improving health equity within dermatology.

## Abbreviations

SOC: skin of color
